# Preoperative diagnosis of hydatid cyst of the breast: a case report

**DOI:** 10.11604/pamj.2013.14.99.2396

**Published:** 2013-03-12

**Authors:** Ali Alamer, Asim Aldhilan, Dorothy Makanjuola, Abdulmohsen Alkushi

**Affiliations:** 1 Medical Imaging Department King Abdul Aziz Medical City for National Guard, Riyadh 11426, P.O Box 22490, Saudia Arabia; 2 Pathology Department 4, King Abdul Aziz Medical City for National Guard, Riyadh 11426, P.O Box 22490, Saudia Arabia

**Keywords:** Hydatid disease, Hydatid cyst, breast lump, mammography, ultrasound, CT

## Abstract

Hydatid cyst of the breast is endemic in some areas like sheep-raising countries. The location of the disease is mostly in the liver and lungs. We presents a case of 66-year-old female with hydatid cyst of the breast diagnosed pre-operatively by core needle biopsy. Complete radiology workup are also provided which includes mammography, ultrasound, and computed tomography images. Hydatid cyst of the breast is extremely rare even in endemic areas, its only accounts for 0.27% of all cases. Only few reports are published in the literatures about breast hydatid cyst and majority of cases have been diagnosed post-operatively with no complete radiology workup.

## Introduction

Hydatid disease is endemic in some areas like sheep-raising countries. *Echinococcus granulosus* is the most common cause of hydatid disease in humans. The location is mostly in the liver (75%) and lungs (15%), with only 10% occurring in other parts of the body. Hydatid disease of the breast is extremely rare even in endemic areas, its only accounts for 0.27% of all cases. Patients usually presents to the hospital with a palpable and painless lump in the breast. It's challenging to differentiate it from other tumors. Only few reports are published and majority of the reported cases have been diagnosed postoperatively. We report a case of hydated cyst of the breast diagnosed preoperatively by core needle biopsy with no complication. Full radiology workup is also provided which includes mammography, ultrasound, and computed tomography (CT) images.

## Patient and observation

A 66-year-old female patient known case of hypertension and diabetic mellitus presented to our institute with palpable mass in the left breast associated with pain of long duration. There was no nipple discharge, or fever. No history of breast trauma, hormone replacement therapy, or family history of breast cancer. On physical examination, large palpable mass is identified within the left breast with regular borders. The nipple, areola, and skin are unremarkable. There was no palpable lymph node in the left axilla. The right breast and axilla were normal and systemic examination did not show any abnormality. Investigations showed normal complete blood count, and chemistry apart from raised serum glucose level. Liver function test is within normal limits. Chest x-ray is grossly unremarkable.

**Figure 1:**
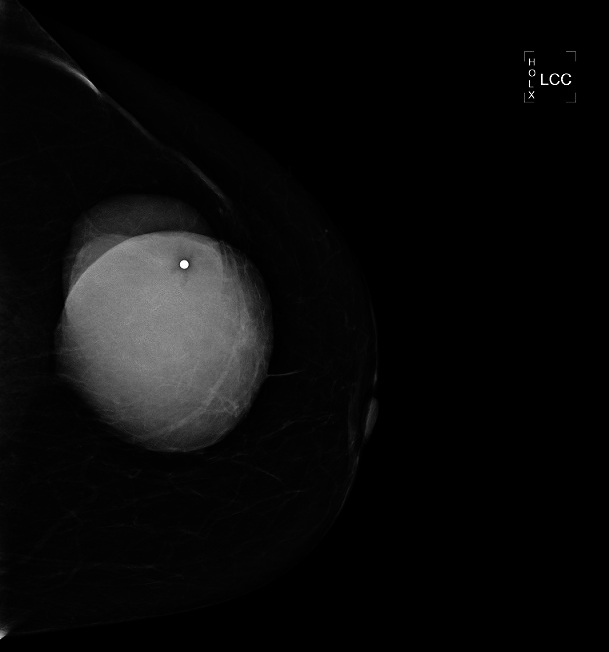
Crainocaudal mammogram image of the left breast shows a large dense mass in the left upper outer quadrant

**Figure 2:**
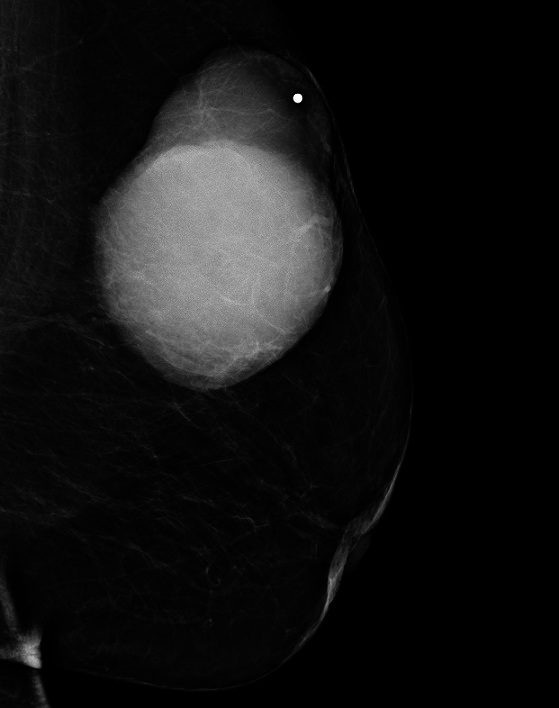
Mediolateral oblique mammogram image of the left breast shows a large dense mass in the upper outer quadrant

On her mammograms, large oval and dense mass is identified within upper outer quadrant of the left breast (Figure 1, Figure 2). The mass is multilobulated and well circumscribed in its contours. It measures about 9.2x7.5 cm. There were no associated microcalcifications. The nipple-areola and skin complex are grossly unremarkable. No significant enlarged axillary lymph nodes are seen. The right breast is within normal limits. Our impression based on the mammograms is unusual large dense mass of the left breast coded as BIRAD 4A taking into consideration the patient age for further evaluation by target ultrasound. The differential diagnosis would include phyllodes tumor, PASH (Pseudoangiomatous stromal hyperplasia), and well circumscribed carcinoma.

**Figure 3:**
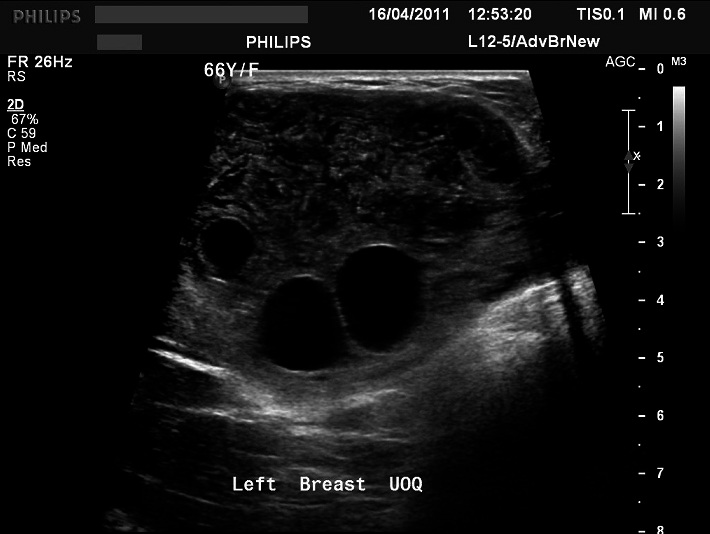
Target ultrasound image of the left breast upper outer quadrant shows a large mass with multiple internal anechoic cysts

**Figure 4:**
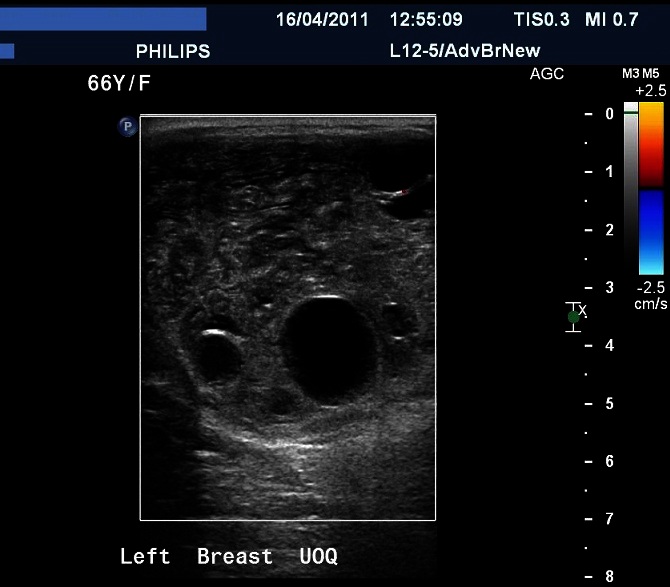
Doppler integrated image shows no internal flow within the mass

**Figure 5:**
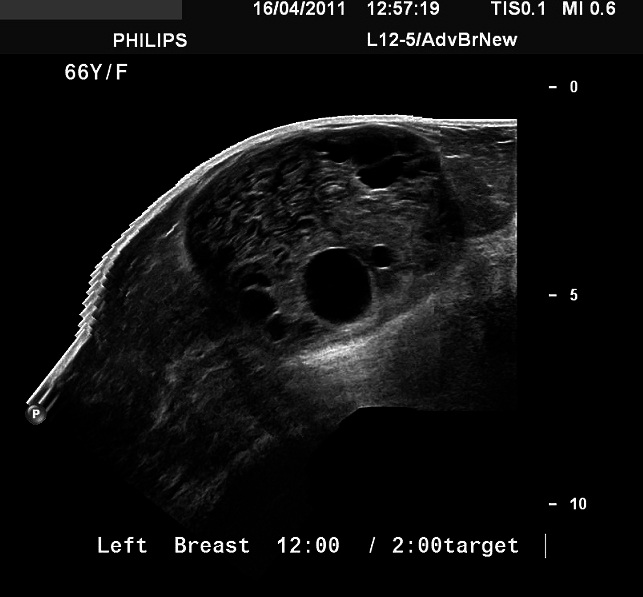
Panoramic ultrasound image of the left breast mass

Ultrasound was performed in same sitting for further characterization using Philips IU22 ultrasound machine. The ultrasound revealed a large heterogeneous and lobulated mass seen in the left breast upper outer quadrant corresponding to the mammographic density. Few internal anechoic cysts are seen at the peripheral aspect of the lesion. Doppler interrogated images were also obtained which revealed no internal vascularity within the mass (Figure 3, Figure 4, and Figure 5). There were no associated enlarged axillary lymph nodes. Plan was made for histological confirmation by biopsy. The procedure risks and benefits were completely explained to the patient. Procedure consent form was obtained from the patient. Ultrasound guided core needle biopsy was performed. The specimen was sent to the pathology department for analysis. The procedure was uneventful. No complications were reported.

**Figure 6:**
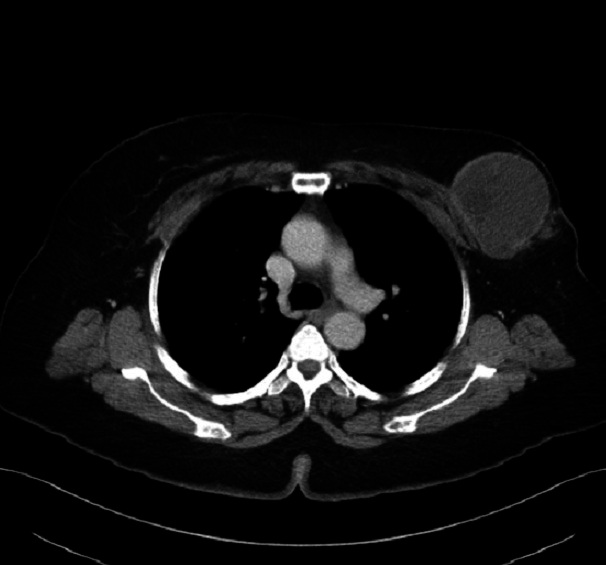
Axial CT image shows left breast mass with internal septations and peripheral enhancement

**Figure 7:**
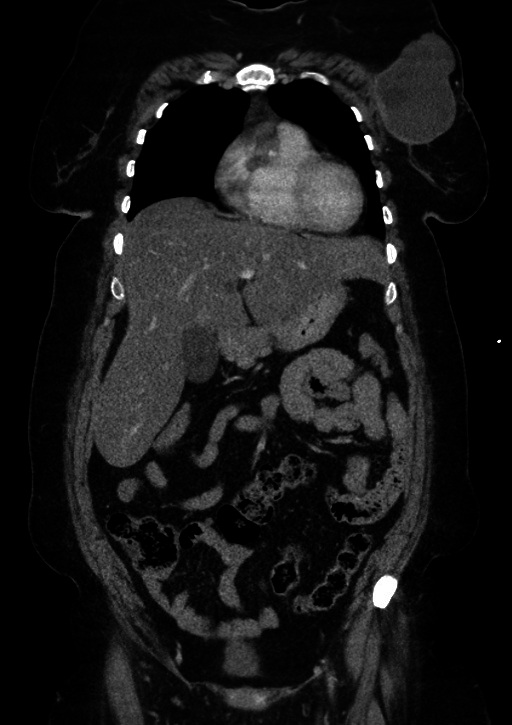
Coronal CT image shows the left breast mass with internal hypo dense cystic component

CT scan was performed for further assessment which showed a large oval hypodense mass in the left breast. Very tiny and thin internal septations were also evident. Peripheral enhancement was seen. No internal enhancement within the mass was identified. The mass measured about 6.8x6.2x9 cm in maximum dimensions (Figure 6, Figure 7). No axillary lymph nodes were noted. The rest of intra thoracic and abdominal structures were within normal limits. The pathology report revealed scanty material formed of fragmented eosinophilic membranes with laminated appearance in keeping with hydatid cyst. The final diagnosis was made to be hydatid cyst of the breast. The patient was referred to the surgery department for surgical resection.

## Discussion

Hydatid disease is parasite infection caused by several species of Cestode Echinococcus but mostly by larva tapworm *Echinococcus granulosus*. Hydatid disease of the breast is extremely rare even in endemic areas; it can be the only primary site or part of disseminated hydatidosis [1]. 60% of hydatid cysts are found in the liver, 30% in the lungs, 2.5% in the kidneys, 2.5% in the heart, 2% in the bone, 1.5% in spleen, 1% in the muscle, and 0.5% in brain [2]. However, in the breast its only accounts for 0.27% of all cases [3].

Typically the patients present with painless breast lump which increase in size over time. It generally affects women between age of 30 and 50 years old; although ages from 20 to 74 years have been reported [4]. When secondary infection occurs, hydatid cyst can′t be distinguish from breast abscess, clinically or by mammography [5].

The screening modalities for diagnosis of breast hydatid disease are mammography, ultrasound, and magnetic resonance imaging (MRI). However, for classification of the cysts ultrasound considered the best choice. Mammography shows a non-specific, homogeneous, smooth, circumscribed lesion. The differential diagnosis based on mammography would includes cyst, fibroadenoma, phylloides tumour and, rarely, circumscribed carcinoma. The characteristic ring shaped structure inside a mass in over penetrated view strongly suggests hydatid cyst. Vega et all noted that the presence of ring shaped structures and interseptal bands in the slowly developing breast mass should suggest a hydatid cyst [6].

The ultrasound findings vary according to the degree of maturation and the complications. Separation of the ruptured endocyst layer from the ectocyst leads to a free floating membrane which produces the so called water lily sign. Hydatid sand composed of hooklets, membrances, and debris give internal echo, and the level of fluid can be seen. The presence of a thicker and more laminated wall, relative to a simple cyst, and a thin calcification layer within the lesion on ultrasonography favors hydatid cyst. In addition, the presence of a cyst in another organs together with a multilocular cystic lesion showing a fluid level in the breast suggests a hydatid cyst [5]. Gharbi et al. [7] have described five types of ultrasound findings for hydatid cysts, including pure fluid collection (type I), fluid collection with a split wall (type II), fluid collection with septa (type III), heterogeneous echo patterns (type IV) and reflecting thick walls (type V).

MRI findings can be helpful but not specific. The findings of cystic lesion with capsular enhancement are suggestive of hydatid cyst. The capsular enhancement is more typical with secondary infection. Hydatid cyst can presents with no capsular enhancement. Diagnosis is frequently delayed due to no specific signs are found at the time of examination, and they instead mimic other pathologies. A hydatid cyst is usually not included in the differential diagnosis of breast lumps due to its rarity, even in endemic areas. Due to the rarity of this condition, the above-mentioned mammographic and sonographic appearances of breast hydatid disease are frequently missed until an operative diagnosis has been made [4]. Rarely, a preoperative diagnosis can be made using a combination of clinical, imaging and fine needle aspiration cytology (FNAC) findings. Serologic tests may be used to confirm the diagnosis and in follow up. A positive serum reaction may occur even in absence of liver and lung involvement [5]. Scoleces, hooklets, and laminated membranes can be identified in FNAC. No urticarial or anaphylactic reactions have been reported as a complication of this procedure. Therefore, FNAC can provide a safe, fast, inexpensive preoperative diagnosis and allow the planning of a cystectomy, minimizing the risk of intraoperative rupture [4]. A hydatid cyst is treated with total excision without any spillage and its recurrence is very rare. Albendazole treatment has been shown to reduce the incidence of recurrence [5, 8].

## Conclusion

Hydatid disease of the breast is extremely rare disease, but it should be considered as a differential diagnosis of breast lumps for individuals residing in endemic areas. Breast mass of long duration without internal flow by ultrasound Doppler images can suggest hydatid cyst. The diagnosis usually made postoperatively. However, ultrasound guided core needle biopsy is safe and good preoperative diagnostic tool.
